# PACAP Protects Adult Neural Stem Cells from the Neurotoxic Effect of Ketamine Associated with Decreased Apoptosis, ER Stress and mTOR Pathway Activation

**DOI:** 10.1371/journal.pone.0170496

**Published:** 2017-01-26

**Authors:** Shiva Mansouri, Ingrid Agartz, Sven-Ove Ögren, Cesare Patrone, Mathias Lundberg

**Affiliations:** 1 Department of Clinical Science and Education, Södersjukhuset, Karolinska Institutet, Stockholm, Sweden; 2 Department of Psychiatric Research, Diakonhjemmet Hospital, Oslo, Norway; 3 Department of Clinical Neuroscience, Karolinska Institutet, Stockholm, Sweden; 4 NORMENT, KG Jebsen Centre for Psychosis Research, Division of Mental Health and Addiction, Oslo University Hospital & Institute of Clinical Medicine, University of Oslo, Norway; 5 Centre for Psychiatry Research, Department of Clinical Neuroscience, Karolinska Institutet, Stockholm, Sweden; 6 Department of Neuroscience, Karolinska Institutet, Stockholm, Sweden; 7 Department of Clinical Science and Education, Södersjukhuset, Internal medicine, Karolinska Institutet, Stockholm, Sweden; Temple University School of Medicine, UNITED STATES

## Abstract

Ketamine administration is a well-established approach to mimic experimentally some aspects of schizophrenia. Adult neurogenesis dysregulation is associated with psychiatric disorders, including schizophrenia. The potential role of neurogenesis in the ketamine-induced phenotype is largely unknown. Recent results from human genetic studies have shown the pituitary adenylate cyclase-activating polypeptide (PACAP) gene is a risk factor for schizophrenia. Its potential role on the regulation of neurogenesis in experimental model of schizophrenia remains to be investigated. We aimed to determine whether ketamine affects the viability of adult neural stem cells (NSC). We also investigated whether the detrimental effect mediated by ketamine could be counteracted by PACAP. NSCs were isolated from the subventricular zone of the mouse and exposed to ketamine with/without PACAP. After 24 hours, cell viability, potential involvement of apoptosis, endoplasmic reticulum (ER) stress, mTOR and AMPA pathway activation were assessed by quantitative RT-PCR and Western blot analysis. We show that ketamine impairs NSC viability in correlation with increased apoptosis, ER stress and mTOR activation. The results also suggest that the effect of ketamine occurs *via* AMPA receptor activation. Finally, we show that PACAP counteracted the decreased NSC viability induced by ketamine *via* the specific activation of the PAC-1 receptor subtype. Our study shows that the NSC viability may be negatively affected by ketamine with putative importance for the development of a schizophrenia phenotype in the ketamine induced animal model of schizophrenia. The neuroprotective effect via PAC-1 activation suggests a potentially novel pharmacological target for the treatment of schizophrenia, *via* neurogenesis normalization.

## Introduction

Today, we recognize that in the adult mammalian brain, a proliferating population of neural stem cells (NSCs) generate new neurons throughout the adulthood life *via* a mechanism known as adult neurogenesis [1, 2]. This process occurs in the subgranular zone (SGZ) of the hippocampus, which supplies new granule cells to the dentate gyrus (DG) of the hippocampus [3] and in the subventricular zone (SVZ) of the lateral ventricle, which provides cellular turnover in the olfactory bulb (OB) in the rodent brain [4] and in the striatum in the human brain [5].

From a functional perspective, previous research has shown that adult neurogenesis plays an important role in several brain functions including plasticity, memory and olfactory functions [6]. In addition, a body of evidence indicates that impaired adult neurogenesis may also be involved in the pathogenesis of various neurological and mental disorders including Alzheimer’s disease (AD), Parkinson’s disease (PD), depression, stroke and schizophrenia [7–10]. In particular, impaired SVZ-neurogenesis in AD animal models [11] and *post-mortem* tissues from human AD brains [12] has been reported and reduced OB neurogenesis in PD animal models has been demonstrated [8, 13].

Schizophrenia is a devastating mental disease strongly affecting cognition and perception [14]. Interestingly, several studies have shown that memory and olfactory dysfunction in schizophrenic patients may be associated with impaired SVZ and hippocampal neurogenesis [15–17]. Due to the complexity to study the role of neurogenesis in human schizophrenia, a number of animal models have been employed [18–20]. One of the most well established animal models for schizophrenia is based on the administration of ketamine [18, 21, 22]. Ketamine is a non-competitive N-methyl-D-asparte (NMDA) receptor antagonist that has been shown to induce symptoms in rodents similar to those associated with schizophrenia in humans (21, 22). How ketamine ultimately mediates its bioactivity has not been fully elucidated but it appears to be dependent on α-amino-3-hydroxy-5-methyl-4-isoxazolepropionic acid (AMPA) receptor activation and on the activation of the mammalian target of rapamycin (mTOR) pathway [23, 24]. Previous studies have shown that ketamine induce neurotoxicity *in vitro*, depending of cell type, dose and exposure time [25–27]. For instance, within a population of cells including human neurons, cortical neural stem cells, embryonic stem cells and neuroblastoma cell lines, significant neurotoxicity has been observed in the range of 100–3000 μM with an exposure time of 24 hours, where human neurons required the highest concentration [25–27]. Neurotoxic effects have also been observed in human clinical settings [28]. However, the direct effect of ketamine on primary adult NSCs is unknown.

How different factors could regulate adult neurogenesis in schizophrenia and animal models of this disease is also poorly understood and remains to be studied. Adult neurogenesis may be regulated by a wide range of factors including growth factors, peptides, exercise and environmental enrichment [9]. The pituitary adenylate cyclase activating polypeptide (PACAP) plays several roles in neurobiological functions, *via* three heptahelical G-protein-linked receptors; PAC1, VPAC1 and VPAC2 [29]. We and others have previously shown that PACAP regulates adult NSCs proliferation and differentiation under normal and toxic conditions [29–33]. Neuroprotective effects of PACAP against ischemia, neurodegeneration and spinal cord injury have also been reported [34–37]. Moreover, previous studies have shown that mice lacking PACAP or its specific receptor (PAC-1) exhibit behavioral abnormalities such as reduced anxiety-like behavior and abnormal social behavior as well as impairment of hippocampal long-term potentiation (LTP) [38–42]. These results indicate that PACAP signaling *via* PAC-1 has a critical role in the development and functional neural pathways that might play an important role in neuropsychiatric disorders [43], perhaps through the regulation of adult neurogenesis.

According to previous genomic studies, the role of PACAP in schizophrenia has shown contradictory results [44, 45]. However, in a recent subsequent functional genomics study in schizophrenic patients, PACAP (ADCYAP1) was identified as a top risk gene to be involved in schizophrenia [46] suggesting a potential important role for PACAP in the pathogenesis of schizophrenia.

The present study was conducted to determine the potential effects of ketamine on the viability of primary adult NSCs isolated from SVZ of the mouse brain and to clarify putative cellular pathways for ketamine activity in these cells. Moreover, we determined the potential *in vitro* efficacy of PACAP to counteract the effects induced by ketamine on primary adult NSCs.

## Methods

### NSCs isolation and cell cultures

The SVZ of the lateral brain ventricles of adult male mice 6 weeks of age (five C57 BL6/SCA mice in each experiment) was micro-dissected by using a micro-dissector scissor and enzymatically dissociated in 0.5 mg/ml trypsin, 0.8 mg/ml hyaluronidase and 80 U/ml deoxyribonuclease I (Sigma-Aldrich, St Louis, MO) in DMEM/F12 containing B27 supplement, 4.5 mg/ml glucose, 100 U/mL penicillin, 100 μg/ml streptomycin sulfate and 12.5 mM HEPES buffer solution (Invitrogen, Stockholm, Sweden). The enzymatic digestion was carried out at 37°C for 20 min. After a gentle trituration with a pipette and mixing, cells were passed through a 70 μm strainer (BD Biosciences, Stockholm, Sweden) and pelleted at 1,000 rpm for 12 min. The centrifugation step was repeated once more after removing the supernatant by adding fresh cold DMEM/F12. The supernatant was then removed, and cells were re-suspended in DMEM/F12 supplemented with B27 and 18 ng/ml human epidermal growth factor (EGF) (R&D systems, Oxon, U.K.). Cells were plated in a 10 cm Petri dish and incubated at 37°C for 7 days in order for neurospheres (NS) to be developed. After 7 days, the NS were collected and centrifuged at 1,000 rpm for 10 min. NS were re-suspended in 0.5% trypsin/EDTA (Invitrogen, Stockholm, Sweden), by incubating at 37°C for 2 min and triturated gently to aid dissociation. After a further 2 min incubation at 37°C, the cell preparation was diluted 1/20 in DMEM/F12 at 37°C. Cells were then pelleted at 1,000 rpm for 10 min and re-suspended in fresh DMEM/F12 containing 18 ng/ml EGF and 16 ng/ml human basic fibroblast growth factor (bFGF) (R&D systems, Oxon, U.K.) before plating. NS were passed every 5 days for 4 weeks and all experiments were performed between passage 2 and 8. Previous work by our group has validated the NSC culture [47].

### Ketamine medium

The sensitivity of mouse adult NSCs for induction of ketamine neurotoxicity have not been previously determined. To examine whether neurotoxicity was dose-dependent, cells were exposed to increasing doses [400 μM –1 mM] of s-ketamine in DMEM/F12 supplemented with B27 and 0.01 ng/ml of EGF.

### ATP assay

Previous reports have indicated that intracellular ATP levels correlate to cell numbers [48]. To measure NSC viability, NSCs were plated as single cells (see above) into 96-well-plates (Corning B.V. Life Sciences, Amsterdam, Netherlands) at the final concentration of 50,000 cells/well with DMEM supplemented with B27 and low EGF. To note, the supplementation of EGF has been adjusted to a minimum level, to produce an experimental condition, where no proliferation is occurring (including in the control cells and PACAP alone treated cells). The rational for this was to produce an assay aimed to study cellular protection in absence of proliferation. 100 nM of PACAP 38 (Phoenix Pharmaceuticals, Burlingame, CA, USA), 30 μM Maxadilan-4 (Max4; PAC-1 specific agonist; graciously provided by R.G. Titus) and 10μM of 2, 3-dihydroxy-6-nitro-7sulfamoyl-benzo[f]quinoxaline-2, 3-dione (NBQX) a selective AMPA receptor inhibitor (R&D systems) were administered with ketamine treatment. After 24 hours of incubation at 37°C (5% CO_2_, 98% humidity), intracellular ATP levels were measured using the Cellular ATP Kit HTS according to the manufacturer’s instructions (BioThema, Stockholm, Sweden). In these experiments, the effect of each treatment at a certain concentration was between 4–8 samples in 3–5 different sets of experiments.

### Western blotting

NSCs were plated as single cells and expanded in a 10 cm Petri dish with EGF/bFGF< (see under cell cultures) for 3–4 days. When NS were formed, the different treatments were added for 24 hours. After exposure, cells were washed with PBS and lyzed in a buffer containing 150mM NaCl, 20mM Tris, 0.1% SDS, 1% Triton X-100, 0.25% Na-deoxycholate, 1mM Na3VO4, 50mM NaF, 2mM EDTA, and Protease inhibitory cocktail (Sigma–Aldrich) on ice for 30 min. Samples were clarified by centrifugation. The supernatants were transferred to new tubes and the total protein concentration was determined by Lowry protein assay (Bio-Rad Laboratories, Stockholm, Sweden). Samples were then mixed with reducing SDS-PAGE sample buffer and boiled for 5 min before performing SDS-PAGE. After electrophoresis, proteins were transferred onto polyvinylidene fluoride (PVDF) membranes (Bio-Rad Laboratories). Immunoblot analyses were performed with antibodies against the cleaved form of caspase-3 (1:1000, polyclonal) (Cell Signaling Technology, Danvers, MA, USA), Bcl-2 (1:200, polyclonal) (Abcam, Cambridge, MA, USA) and phosphorylated mTOR (1:2000, monoclonal) (Cell Signaling Technology, Danvers, MA, USA). Immuno-reactive bands were developed using ECL (GE Healthcare, Stockholm, Sweden), imaged with a GelDoc system or Syngene system and quantified with Quantity One software (Bio-Rad Laboratories). After imaging, to verify equal protein loading, the PDVF membranes were stained with Coomassie blue (Fermentas, St. Leon-Rot, Germany) as illustrated in previous studies [49–51], total mTOR (1:2000, monoclonal) (Cell Signaling Technology, Danvers, MA, USA) or β-actin (1:800, polyclonal) (Santa Cruz Biotechnology. Inc., Germany). In these experiments, the effect of each treatment at a certain concentration was determined in single/double samples in 2–5 different set of experiment.

### Quantitative RT-PCR

To quantify the ER stress-inducible transcription factor CHOP mRNA levels the total RNA was extracted using Aurum total RNA-mini kit (Bio-Rad Laboratories, Stockholm, Sweden) and the RNA was treated with DNase I (Bio-Rad Laboratories, Stockholm, Sweden) to eliminate possible DNA contamination, according to the manufacturer’s protocol. Total mRNA was reversely transcribed into cDNA by using an iScript^™^cDNA Synthesis Kit (Bio-Rad Laboratories, Stockholm, Sweden). The expression levels of mRNAs were measured by SYBR green based quantitative RT-PCR (iQ^™^ SYBR^®^ Green Supermix; Fermentas, St. Leon-Rot, Germany) using mouse-specific primer pairs for CHOP (forwdard:5'-GAAAGCAGAACCTGGTCCAC-3, reverse: 5'-GACCTCCTGCAGATCCTCAT-3´) (Invitrogen, Stockholm, Sweden). β-actin was used as an internal standard.

### Statistical analysis

The differences between groups were tested with one-way ANOVA followed by post hoc Fisher LSD test or Kruskal-Wallis followed by Dunn’s test if data were not normally distributed. All statistical analyses were performed using Sigma Plot software v. 11. Data are presented as mean ±SEM. *P* < 0.05 was considered statistically significant.

All experiments were conducted according to the regional ethics committee for animal experimentation conforming to the "Guide for the Care and Use of Laboratory Animals" published by U.S. National Institutes of Health (NIH publication # 85–23, revised 1985). The C57 BL6/J mice were imported from Nova-SCB, Stockholm, Sweden. The regional ethical committee (Stockholm Södra djurförsöksetiska nämnd) has approved all animals studies presented in the manuscript. Animals were sacrificed in CO_2_ chamber and cervical dislocation.

## Results

### Ketamine impairs NSC viability in a dose-dependent manner

**Fig 1:**
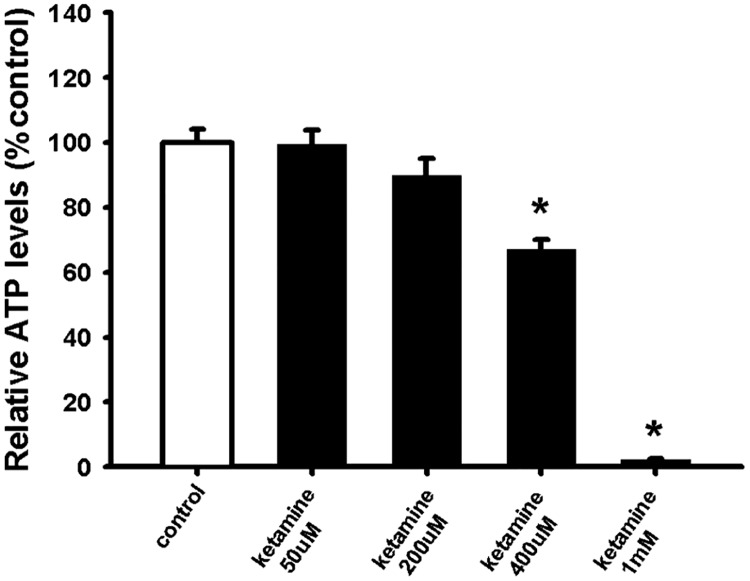
Ketamine decreases NSC viability in a dose-dependent manner. NSCs were plated as single cells treated with 50 μM, 200 μM, 400 μM and 1 mM ketamine. To measure cell viability, intracellular ATP levels were measured after 24 hours. Data are shown as mean ±SEM (n = 22–35). Kruskal-Wallis followed by Dunn’s test was used. Differences were considered significant at P <0.05. * denotes P<0.05 compared with control.

To study the effect of ketamine on NSC viability, different concentrations of ketamine [50 μM, 200 μM, 400 μM and 1 mM] were added to NSCs isolated from the SVZ of the adult mouse. NSC viability was assessed after 24 hours by measuring intracellular ATP levels. The results show that 400 μM and 1 mM ketamine significantly impaired NSC viability in a dose-dependent manner (Fig 1). Previous studies have reported significant neurotoxicity in the range of 100–3000 μM with the same exposure time [25–27]. We selected a ketamine dose of 400 μM for the subsequent tests due to its significant and submaximal effect on cell viability.

### Ketamine decreases NSC viability *via* AMPA receptor activation

**Fig 2:**
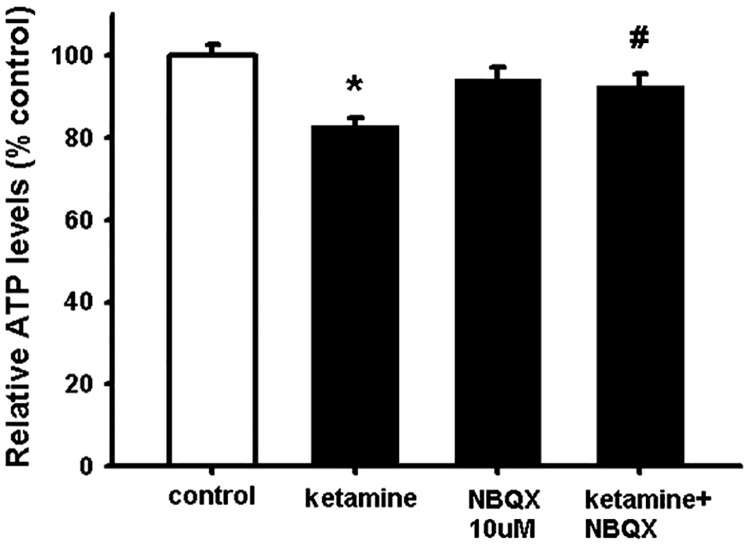
Ketamine impairs NSC viability *via* AMPA receptor activation. NSCs were plated as single cells and treated with 400 μM ketamine or pre-treated with 10 μM NBQX prior to 400 μM ketamine exposure. After 24 hour incubation, intracellular ATP levels were measured. Data are shown as mean ±SEM (n = 23–39). Kruskal-Wallis followed by Dunn’s test was used. Differences were considered significant at P<0.05. * denotes P<0.05 compared with control, # denotes P<0.05 compared to ketamine.

In order to understand the potential mechanism at the basis of the toxic ketamine effect on NSCs, we pre-treated NSCs with the selective and competitive AMPA receptor antagonist NBQX before ketamine exposure. The results show that NBQX significantly suppressed the ketamine-induced cell death indicating that the toxic effect by ketamine on NSC is mediated by AMPA receptor activation (Fig 2).

### PACAP counteracts ketamine-induce neurotoxicity *via* PAC-1 activation

**Fig 3:**
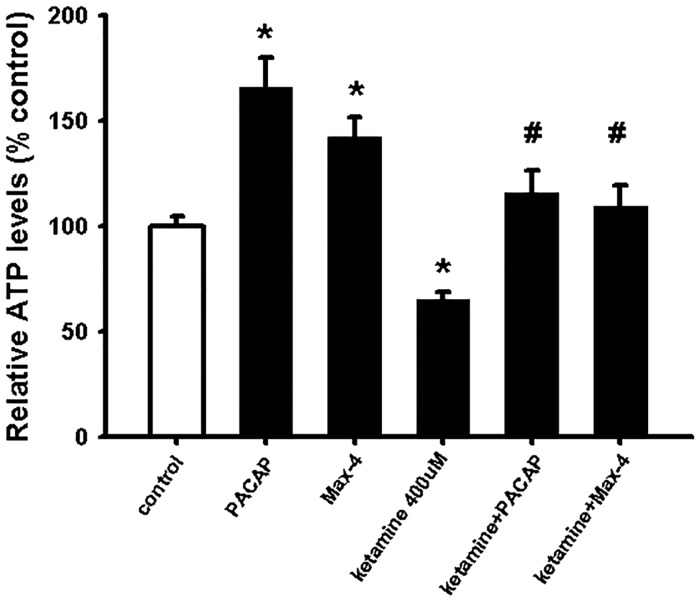
Ketamine-induced cell death is counteracted by PACAP *via* PAC-1 receptor activation. NSCs were plated as single cells. Cells were incubated with 400 μM ketamine and PACAP (100 nM) or agonist for PAC-1; Max-4 (30 nM). After 24 hour incubation, intracellular ATP levels were measured for cell viability. Data are shown as mean ±SEM (n = 13–39). Kruskal-Wallis followed by Dunn’s test was used. Differences were considered significant at P<0.05. * denotes P<0.05 compared with control, # denotes P<0.05 compared to 400 μM ketamine.

To determine whether PACAP had a neuroprotective effect against ketamine, we treated NSCs with 100 nM PACAP alone or with 400 μM ketamine. NSCs viability was determined 24 hours after. The result in Fig 3 shows that PACAP increased NSC viability under normal conditions as previously reported [30, 31]. Moreover, PACAP was able to counteract completely the negative effect of ketamine on NSCs (Fig 3).

Previous studies have revealed that NSCs expresses the PACAP receptors PAC-1 and VPAC-1 *in vitro* [30]. In a previous study, we showed that a decreased NSC viability, induced by palmitate, was counteracted by PACAP *via* activation of PAC-1 receptor [30]. Therefore, to determine whether the protective effect of PACAP against ketamine was also mediated by PAC-1 activation, NSCs were treated with the specific agonist for PAC-1; Max-4 (30 nM) [52], with 400 μM ketamine for 24 hours. The results in Fig 3 show that Max-4 was able to counteract entirely the negative ketamine effect on NSCs viability, similarly to PACAP.

### PACAP counteracts impaired NSC viability by ketamine in correlation with decrease apoptosis

**Fig 4:**
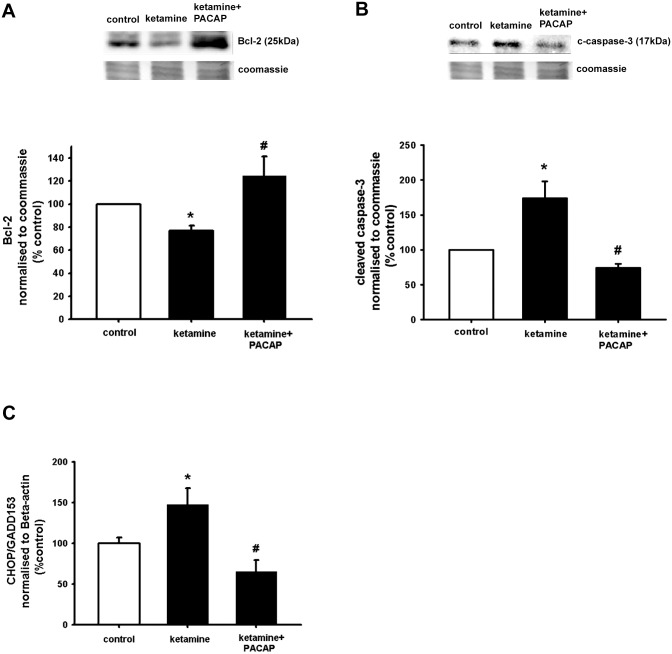
PACAP counteracts ketamine induced apoptosis and ER stress. NSCs were plated as single cells and treated with 400 μM ketamine alone or with 100 nM PACAP and 400 μM ketamine. After 24 hours incubation, cells were harvested for Western blot analysis **(A, B)** or harvested for mRNA extraction and quantitative RT-PCR experiments **(C)**. To obtain quantitative measurements Bcl-2 protein levels and cleaved caspase-3 were normalized against coomassie blue. Data are shown as mean ±SEM (A, n = 5–6; B, n = 3–5, C, n = 4–6). Kruskal-Wallis followed by Dunn’s test. Differences were considered significant at P<0.05. * denotes P<0.05 compared with control, # denotes P<0.05 compared to 400 μM ketamine.

Having demonstrated that PACAP was able to counteract the decreased NSCs viability by ketamine, we tested whether this effect occurred in correlation with markers related to apoptosis. NSCs were plated as single cells and treated with 400 μM ketamine alone or treated with 100 nM PACAP and 400 μM ketamine. After 24 hour incubation, cells were harvested for Western blot analysis to assess the levels of the anti-apoptotic protein Bcl-2 and the pro-apoptotic cleaved form of caspase-3 by Western blotting analysis. The results showed that this dose of ketamine induced a significant decrease of Bcl-2 protein levels (Fig 4A) and an increase of the protein levels of the cleaved form of caspase-3 (Fig 4B). Moreover, as shown in Fig 4A and 4B, PACAP was able to significantly increase protein levels of Bcl-2 and to decrease cleaved caspase-3 protein expression. Similar results were obtained with β-actin normalization (S1 Fig). These data indicate that PACAP have an activity to increase cell viability alone and to counteract ketamine induce neurotoxicity.

### PACAP counteracts ketamine-induce neurotoxicity in correlation with decreased ER stress

ER stress plays an important role in the development and pathology of neurodegenerative diseases [53]. To study whether ketamine induces ER stress, NSCs were exposed to 400 μM of ketamine. After 24 hours, quantitative RT-PCR for the mRNA levels of the ER stress marker CHOP/GADD153 [54] was performed. The result in Fig 4C shows that CHOP/GADD153 was significantly up-regulated in the presence of 400 μM ketamine in comparison to control.

To study the effect of PACAP on ER stress we treated NSCs with PACAP and 400 μM ketamine. We show that PACAP was able to abolish the effect of ketamine on increased CHOP/GADD153 mRNA levels (Fig 4C). In summary, these data indicate that PACAP increase NSC viability alone and against ketamine induce neurotoxicity with an association of decreased ER stress and apoptosis.

### PACAP counteracts impaired NSCs viability by ketamine in correlation with the inhibition of mTOR pathway

**Fig 5:**
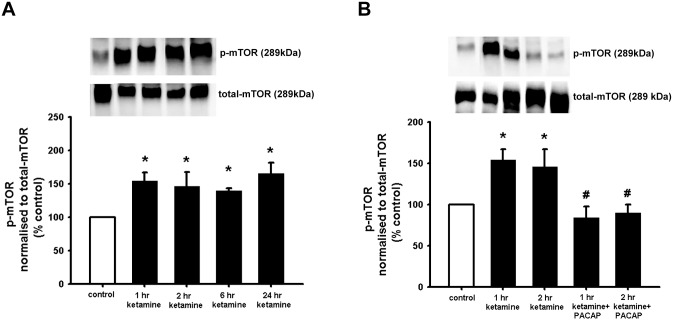
PACAP counteracts ketamine induced mTOR activation. NSCs were plated as single cells and treated with 400 μM alone or with 100 nM PACAP and 400 μM ketamine. Cells were treated as indicated and after 1, 2, 6 and 24 hour incubation cells were harvested for Western blot experiments **(A)**. Cells were treated as indicated and incubated for 1 and 2 hours and harvested for Western blot experiments **(B)**. To obtain quantitative measurements p-mTOR were normalized against total-mTOR. Data are shown as mean ±SEM (A, n = 4–8; B, n = 3–5). Kruskal-Wallis followed by Dunn’s test or Fisher LSD test was used. Differences were considered significant at P<0.05. * denotes P<0.05 compared with control, # denotes P<0.05 compared to ketamine + 1 hour or ketamine + 2 hours.

The mechanistic target of rapamycin (mTOR) signalling pathway plays a central role as a regulator of cell growth, protein synthesis, gene expression, and metabolic balance [55]. Due to its important function in the nervous system, we decided to analyse the mTOR activity in NSCs in relation to observed effects of ketamine and PACAP on NSC viability. To determine whether mTOR was affected by ketamine-induce cell death, NSCs were treated with 400 μM ketamine for up to 24 hours (1, 2, 6, 24 hr). The results in Fig 5A show that ketamine induced a rapid increase of mTOR phosphorylation after 1-hour incubation, an activity that remained over the time period tested. We also tested the putative activity of PACAP to inhibit ketamine induced mTOR activation. The results show that PACAP was able to significantly decrease the expression of p-mTOR at 1 and 2 hours of ketamine treatment, as shown in Fig 5B.

## Discussion

**Fig 6:**
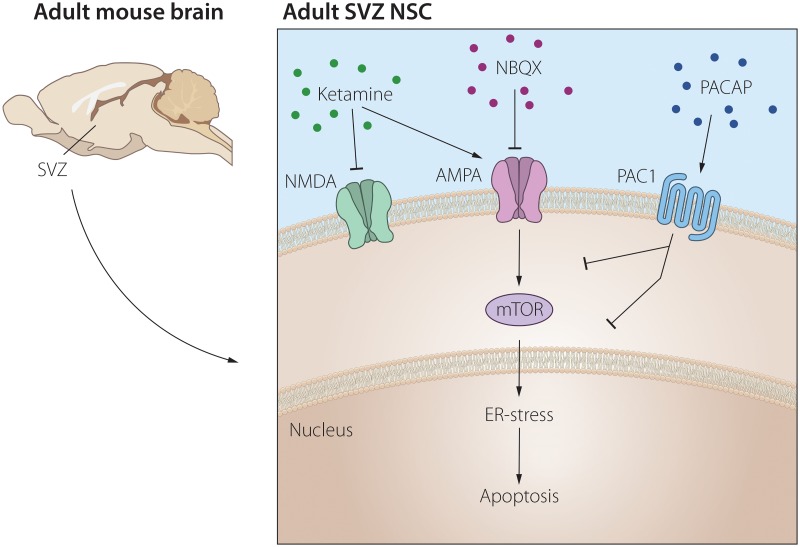
A schematic diagram showing a proposed signaling pathway for PACAP mediated suppression of ketamine induced neurotoxicity of NSCs *in vitro*.

The current study was conducted to explore the potential effects of ketamine on NSCs and to understand the cellular pathways mediated by ketamine. In addition, we determined the possible *in vitro* activity of PACAP to counteract effects on NSCs by ketamine. We show that ketamine dose-dependently, *via* AMPA receptor activation, decreases NSC viability in association with the induction of apoptosis, and ER stress. Moreover, we also show that PACAP, *via* PAC-1 receptor activation, counteracts the impaired NSC viability induced by ketamine, in association with decreased apoptosis, ER stress and in correlation with inhibition of the mTOR signalling pathway (Fig 6).

Our observation of a significant ketamine-induced neurotoxic activity on NSCs at 400 μM are in agreement with previous *in vitro* studies in which sub-toxic concentrations of ketamine (100–3000 μM) have been shown to induce apoptosis in numerous cell types including human embryonic stem cells, cortical neural stem cells, embryonic stem cells and neuroblastoma cell lines (see Intro). Our findings of a neurotoxic effect induced by ketamine are in accordance with animal and human clinical studies [28, 56–58]. However, in the reported *in vivo* studies the concentration of ketamine is always specified, with few exceptions, as the amount of ketamine injected (mg/kg) and not as peak concentration produced in fluids and tissues. This makes translational comparison between *in vitro* and in *vivo* studies difficult. Importantly, in a study by Cohen *et al*, 80 mg/kg of ketamine was given to rats intravenously and the level of ketamine distributed to plasma and brain tissue was analyzed [59]. One minute post injection, a ketamine peak was observed in both plasma and brain tissue, where the plasma and brain tissue concentrations were approximately 45 μM and 360 μM, respectively, i.e. rather near to the concentration used for the present study.

Furthermore, we demonstrated that the ketamine effect was mediated through AMPA receptor activation and was associated with activation mTOR pathway. Previous work by Dong *et al*. [60] showed that altered expression PI3K/Akt signalling by ketamine may be involved in ketamine-induced neurotoxicity in rat fetal neural stem progenitor cells, and that the PI3K/Akt pathway can stimulate the mTOR leading to cell death [61]. In the present study, we observed an association between decreased NSC viability and increased mTOR activity that was counteracted by PACAP. However, we cannot conclude from our experiments whether the increased mTOR activity has a define role for ketamine-induce neurotoxicity. Future studies needs to be conducted to verify the role of mTOR activity e.g. by blocking mTOR activity by using rapamycin.

ER stress and the unfolded protein response (UPR) are activated in several neurodegenerative diseases, and prolonged ER stress leads to apoptosis [53, 62]. Interestingly, recent studies have revealed abnormal ER function in damaged neurons following treatments used for schizophrenia-related injuries such as NMDA receptors antagonist and methamphetamine [63]. Here we show that under ketamine exposure, ER stress is activated by the increase of CHOP/GADD153 mRNA levels in NSCs. To our knowledge this is the first study to show that ketamine induces ER stress in primary adult NSCs.

Having shown that ketamine induced neurotoxicity in adult NSCs; we tested the potential activity of PACAP to counteract ketamine effects. PACAP was chosen since it has shown to have neurogenic effects and neuroprotective properties *in vitro* and *in vivo* in several neurodegenerative diseases and disorders [[64, 65] see Intro]. In addition, a recent study by Lamine *et al*. demonstrated that a synthetic analogue of PACAP, with potential to be developed clinically, showed anti-apoptotic and anti-inflammatory effects both *in vitro* and *in vivo* in PD animal models [36]. This may indicate that PACAP may be useful in the treatment for neurodegenerative disorders. Furthermore, neuroprotection through PAC-1 signalling is well documented [34]. Moreover and importantly, it’s been reported that impairment of the PACAP receptor system could contribute to the symptoms associated with schizophrenia [38]. We found that PACAP *via* specific activation of the PAC-1 receptor, significantly enhanced NSC viability under ketamine treatment, by inhibiting apoptosis and ER stress. It’s been reported that over-activation of AMPA receptors leads to cytotoxicity and neuronal death [66] and a study by Costa *et al*. [67] showed that PACAP reduced AMPA-current on CA1 pyramidal neurons. This suggests that the neuroprotective effects reported in our study by PACAP may be achieved by blocking the negative AMPA receptor activation on NSCs, although this role should be further analysed by measuring the level of ER stress and apoptosis in the presence of AMPA receptor antagonist (NBQX).

Although speculative, our results of impaired NSC viability by ketamine might indicate that one of the mechanisms by which schizophrenia negatively affects the brain may occur *via* the impairment of adult neurogenesis. To prove this hypothesis, our results will need to be validated *in vivo* by investigating whether disturbed neurogenesis occurs in the OB and striatum of animal model of schizophrenia. Moreover, it will be interesting to test in these animal models the potential activity of PACAP analogues as pharmacological agent to normalize neurogenesis as observed in the recent work by Lamine *et al*. [36].

## Conclusions

In summary, we show that under an acute ketamine treatment, NSCs viability is impaired in correlation with increased apoptosis and ER stress. We also show a significant neuroprotective effect of PACAP *via* PAC-1 receptor activation on NSCs exposed to ketamine that correlated to decreased apoptosis and ER stress; *via* blockade of the mTOR-signalling pathway (Fig 6). We believe that these results should motivate to further address the neuroprotective role of PAC-1 agonists in animal model of schizophrenia aiming to identify efficacious compounds for the treatment of schizophrenia through the modulation of adult neurogenesis.

## Supporting Information

S1 Fig PACAP counteracts ketamine induced apoptosis. NSCs were plated as single cells and treated with 400 μM ketamine alone or with 100 nM PACAP and 400 μM ketamine. After 24 hour incubation, cells were harvested for Western blot analysis **(A, B)**. To obtain quantitative measurements Bcl-2 protein levels and cleaved caspase-3 were normalized against β-actin. Data are shown as mean ±SEM (A, n = 3, B, n = 3–4). Kruskal-Wallis followed by Dunn’s test. Differences were considered significant at P<0.05. * denotes P<0.05 compared with control, # denotes P<0.05 compared to 400 μM ketamine.
(TIFF)

S1 File The ARRIVE Guidelines Checklist. The ARRIVE Guidelines Checklist for reporting *in vivo* experiment in animal research.
(PDF)
