# Supplementary figures and images for: PACAP Protects Adult Neural Stem Cells from the Neurotoxic Effect of Ketamine Associated with Decreased Apoptosis, ER Stress and mTOR Pathway Activation
Source: PLoS One. 2017 Jan 26;12(1):e0170496. doi: 10.1371/journal.pone.0170496 (PMC5268395; doi:10.1371/journal.pone.0170496)

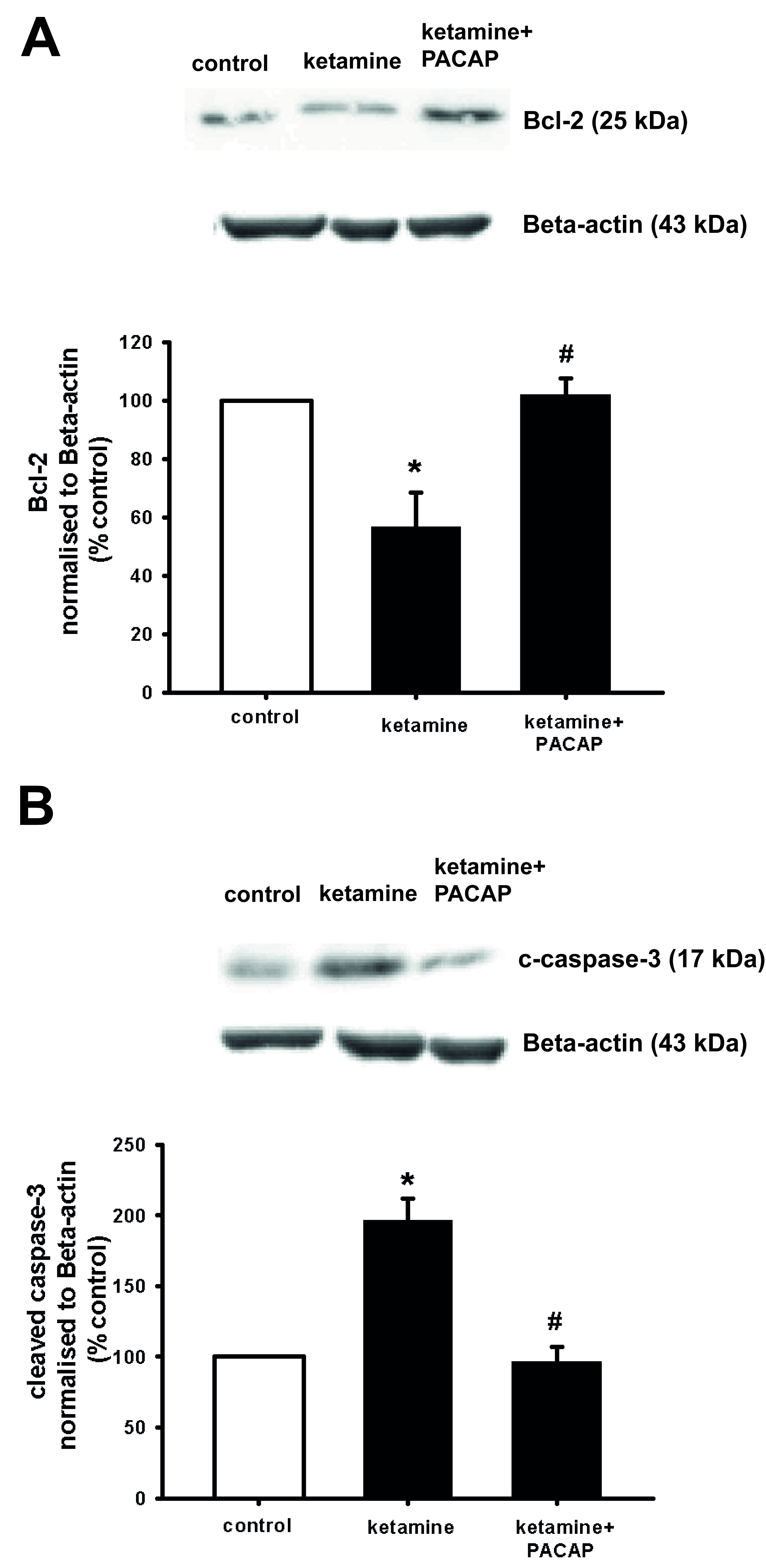

Supplement: S1 Fig — NSCs were plated as single cells and treated with 400 μM ketamine alone or with 100 nM PACAP and 400 μM ketamine. After 24 hour incubation, cells were harvested for Western blot analysis (A, B). To obtain quantitative measurements Bcl-2 protein levels and cleaved caspase-3 were normalized against β-actin. Data are shown as mean ±SEM (A, n = 3, B, n = 3–4). Kruskal-Wallis followed by Dunn’s test. Differences were considered significant at P<0.05. * denotes P<0.05 compared with control, # denotes P<0.05 compared to 400 μM ketamine. (TIFF) [file pone.0170496.s001.tiff]
